# Historical Investigation of Fowl Adenovirus Outbreaks in South Korea from 2007 to 2021: A Comprehensive Review

**DOI:** 10.3390/v13112256

**Published:** 2021-11-10

**Authors:** Jongseo Mo

**Affiliations:** US National Poultry Research Center, Exotic & Emerging Avian Viral Diseases Research, Southeast Poultry Research Laboratory, U.S. Department of Agriculture, 934 College Station Rd., Athens, GA 30605, USA; jongseo.mo@usda.gov

**Keywords:** fowl adenovirus, aviadenovirus, South Korea, epidemiology, HHS, IBH

## Abstract

Fowl adenoviruses (FAdVs) have long been recognized as critical viral pathogens within the poultry industry, associated with severe economic implications worldwide. This specific group of viruses is responsible for a broad spectrum of diseases in birds, and an increasing occurrence of outbreaks was observed in the last ten years. Since their first discovery forty years ago in South Korea, twelve antigenically distinct serotypes of fowl adenoviruses have been described. This comprehensive review covers the history of fowl adenovirus outbreaks in South Korea and updates the current epidemiological landscape of serotype diversity and replacement as well as challenges in developing effective broadly protective vaccines. In addition, transitions in the prevalence of dominant fowl adenovirus serotypes from 2007 to 2021, alongside the history of intervention strategies, are brought into focus. Finally, future aspects are also discussed.

## 1. Introduction

Since the first discovery of fowl adenoviruses (FAdVs) in 1949, for decades, they have been recognized as critical viral pathogens within the poultry sector due to their widespread occurrence and economic implications worldwide [1]. FAdV outbreaks can cause significant economic impacts on the poultry industry, from poor performance and growth retardations to flock mortalities. These isometric non-enveloped viruses belong to the family Adenoviridae, genus Aviadenovirus, and carry a double-stranded DNA genome (dsDNA) with a size of ~45 kb. FAdVs are taxonomically classified into five species (A–E) and twelve (1 to 8a and 8b to 11) serotypes based on their restriction fragment length polymorphism (RFLP) profiles in the hexon gene, which act as subtype-specific antigenic determinants [2,3,4]. In addition, FAdV infections have been confirmed in various avian species other than chickens, such as turkeys, geese, ducks, guinea fowl, pigeons, ostriches, and quails [1].

FAdV infections are associated with a wide range of diseases in birds, such as adenoviral gizzard erosion (AGE) [5], hepatitis-hydropericardium syndrome (HHS) [6] and inclusion body hepatitis (IBH) [7]. AGE features macroscopic lesions in affected gizzards such as inflammation and ulceration, and the majority of outbreaks trace back to FAdV-1 (species A) strains [8,9]. HHS, also known as the hydropericardium syndrome (HPS) is generally caused by FAdV-4 (species C), with infected birds exhibiting accumulation of straw-colored transudates in the pericardial sac, associated with nephrotic and hepatic lesions [10,11]. Lastly, IBH is characterized by a sudden onset of mortality with lesions such as enlarged pale liver and basophilic intranuclear inclusion bodies in hepatocytes [12,13]. FAdV-2, 11 (species D) and FAdV-8a and b (species E) act as main etiologic agents for IBH [1].

Vertical and horizontal transmission are known to be the primary routes of FAdV infection [14,15,16,17]. Due to high cloacal shedding titers in the feces, FAdVs can be horizontally transmitted easily through the oral–fecal route [18]. Horizontal transmission of FAdVs can be facilitated in birds that are associated with immunosuppression due to other viral infections, such as chicken anemia virus (CAV) or infectious bursal disease virus (IBDV) [19,20,21]. Vertical transmission has also been recognized as one of the crucial biological features of FAdVs, and evidence of the virus being transmitted to progenies via embryonated eggs is well documented [15,22,23]. One interesting aspect of vertical transmission of FAdVs is that they can remain latent and undetected for some time and be reactivated in young birds that are immunosuppressed [24,25,26], although vertically transmitted FAdVs are still capable of causing losses in healthy progenies with no co-infections [27]. Depending on the strain’s pathogenicity, cases of vertical transmission can result in significantly decreased weight gain alongside adverse effects on other production parameters, leading to severe economic losses [17]. Moreover, such situations have grave repercussions from the producer’s point of view, since affected birds are unlikely to recover from growth retardation until the harvest age.

Historically, it has been perceived that clinical manifestations of FAdVs were the results of opportunistic infections as a secondary disease in immunosuppressed birds due to other primary viral diseases [28]. However, there is also increasing evidence for FAdVs acting as primary pathogens in the absence of predisposing factors or co-infections with other viruses [13,29,30,31]. Moreover, FAdV-8b and -11, which are known etiologic agents for IBH, were proven to be capable of causing primary diseases in chickens [32]. Maternal antibodies passed down from parental to progeny birds play a significant role in preventing the disease, so effective control measures are typically initiated by vaccination programs. Most involve inactivated vaccines [33], although recently, subunit vaccines that are based on the capsid fiber-2 protein [34,35] or virus-like particles (VLPs) [36] are gaining attention.

Like other viruses from the Adenoviridae family, FAdVs can also cross the species barrier and adapt to host environments, broadening the host range. FAdV infections in wild birds have been widely documented [37,38,39]. Increasing evidence suggests that wild birds may function as FAdV reservoirs and transmit the virus to domestic hosts [39], although interspecies transmission could also occur the other way around from domestic poultry to wild birds [37]. Typically, well-adapted FAdVs exhibit lower levels of virulence in wild birds, but some virulent strains could still cause clear clinical signs [37,40].

There has been an increase in reports of HHS and IBH outbreaks from various regions in the last ten years, such as in Europe [41,42,43,44], Asia [45,46,47,48,49], Oceania [30], North America [50], South America [51,52], and Africa [53,54], indicating even geographical spread of FAdVs. As in most Asian countries, FAdV-induced outbreaks in poultry have been observed in South Korea for many years, and, to date, these outbreaks persist. From 2007 to 2021, a total of 387 clinical cases of FAdVs were officially documented, with an average of 25.8 cases per year, involving 6 serotypes during the 15-year period in South Korea (Table 1). The cases were evenly spread across all eight provinces, and documented mortalities of infected flocks ranged from 0.01% to 55% between 2007 and 2010 [55] but sharply declined after that period to 0.5–4% due to heightened biosecurity and implementation of vaccination programs [45,56,57]. The purpose of this comprehensive review is to provide an overview of the history and status of FAdV outbreaks in South Korea and to update the epidemiological situation that was observed in recent years.

## 2. History of Fowl Adenovirus Outbreaks in South Korea

### 2.1. FAdV Outbreaks in 2007–2012

The first official case of an FAdV in South Korea was reported in 1981 [61]. Documented evidence described the case as “pronounced pathological lesions in the liver swollen with mottled appearance paired with massive hemorrhages and necrosis”, which were typical findings for IBH. However, the molecular characteristics of the pathogens were not determined. Since then, most FAdV case reports in South Korea were made upon clinical examinations until Kim and colleagues [62] first characterized the molecular properties of strains responsible for multiple outbreaks in 2007. Various types of commercial chicken farms (broilers, layers, and breeders) were affected, and postmortem examinations demonstrated that the features of gross and histological lesions were typical of HHS. Further investigations revealed that all outbreaks in this report were attributed to FAdV-4, with the isolates sharing the highest similarities with the Japanese KR5 FAdV-4 reference strain [63,64].

**Figure 1:**
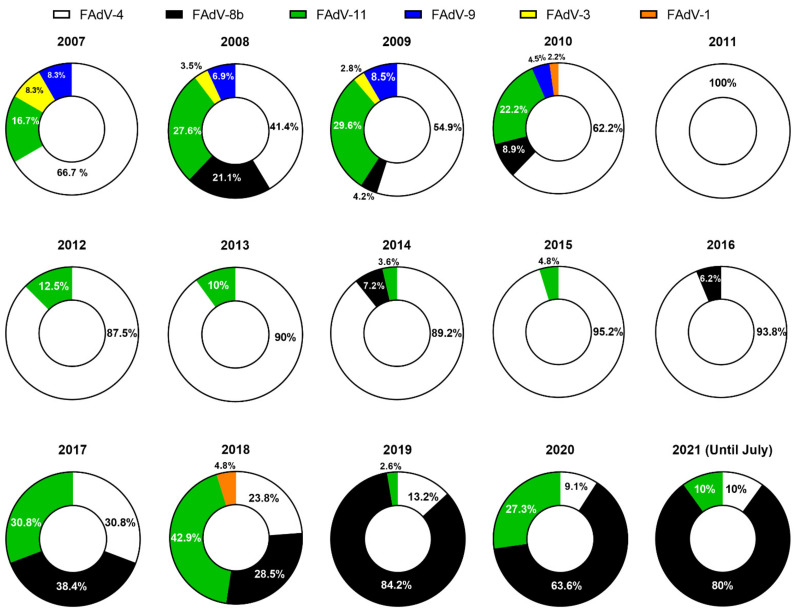
Proportion of reported cases for each FAdV serotype in South Korea from 2007 to 2021. Data are based on published literature [45,55,58,59,60] and unpublished data (Supplementary Materials).

After this report, extensive epidemiologic surveys began to take place. The annual distribution of FAdV cases confirmed by serotype between 2007 and 2012 in South Korea is listed in Figure 1. In 2011, Lim and colleagues isolated and identified fifty-five strains from commercial chicken flocks that had histories of either HHS or IBH from 2007 to 2010 and reported that FAdV-4 and FAdV-11 were the predominant serotypes followed by FAdV-9 and FAdV-3 over that particular time frame [58]. The strains were isolated from all eight provinces of South Korea, indicating even distribution of virus spread, mainly affecting broilers. Interestingly, the prevalence of FAdV-4 and FAdV-11 showed a significant increase starting from 2009, with cases of FAdV-9 sharply declining, making them the two most frequently isolated serotypes in 2010. Among the affected flocks, FAdV-4 was the only single serotype that was primarily responsible for HHS. Investigations of co-infections in FAdV-infected birds were not conducted, although the authors assumed that the live IBDV vaccines might have caused transient immunosuppression in the birds, thus exacerbating FAdV infections [65].

Choi and colleagues carried out a similar study focusing on nationwide FAdV incidents in chicken farms over the same time frame (2007–2010) as reported in Lim’s study [55]. The serotypes of thirty-nine FAdV isolates obtained from clinical cases were determined with investigations focusing on co-infections with other immunosuppressive pathogens. Consequently, it was found that most were broiler cases, with FAdV-4 and -11 as predominant serotypes. However, in this study, outbreaks with FAdV-8b were confirmed instead of FAdV-9 and FAdV-3 as in Lim’s study. Collectively, their findings suggested that FAdV-4 was most commonly associated with HHS, whereas IBH cases were solely attributed to FAdV-8b and -11 in South Korea. Co-infections with other immunosuppressive viral pathogens were also confirmed; CAV infections comprised up to one-third of all clinical FAdV cases, suggesting that they played a significant role in complications associated with immunosuppression. However, standalone FAdV infections comprised nearly half of all cases, also indicating the possible role of FAdVs as self-standing pathogens in the absence of CAV or IBDV infections [29]. Most of the standalone FAdV cases were caused by FAdV-4, which is not surprising, as it is commonly known that some virulent strains from this serotype are capable of acting as primary pathogens that can cause immunosuppression in the hosts by targeting lymphoid tissues and depleting T and B lymphocytes [66,67,68].

Apart from the studies mentioned above, a sporadic case of an FAdV-1 outbreak was reported in 2010 [59], and unpublished data collected from clinical cases that confirmed FAdVs from 2007 to 2012 (Supplementary Table S1 and Figure S1) also showed that FAdV-4 was the predominant serotype, making up 83% (85/103) of all cases, followed by FAdV-11 (14%) and FAdV-8b (3%). Remarkably, the emergence of FAdV-4 was significantly noticeable in 2011, in which all FAdV field cases (100%, 17/17) were attributable to FAdV-4 (Table 1, Figure 1).

### 2.2. Development of Domestic FAdV Vaccines

Vaccination has been perceived as a cost-effective intervention to prevent and control FAdV-induced diseases, particularly with regard to HHS caused by FAdV-4. As it seemed evident that FAdV-4 was the predominant serotype circulating in South Korea in 2007–2012, causing numerous outbreaks of HHS in the poultry sector nationwide, vaccine development efforts to counter this set of emerging viruses were prioritized. Most of these efforts were focused on designing inactivated vaccines, as several related studies have achieved the control of FAdV infection by conferring protection to both immunized flocks and their progenies using inactivated or attenuated vaccines [69,70,71]. The first attempt at developing a domestic FAdV-4 vaccine was made in 2010. A research team selected an FAdV-4 strain isolated in 2008 [62] and prepared an inactivated oil emulsion vaccine, which successfully elicited neutralizing antibodies and protected challenged SPF chickens [56]. Clear seroconversions were evidenced in vaccinated birds with no occurring HHS confirmed in postmortem examinations. Since then, multiple vaccine strains have been selected and tested in trials conducted in South Korea to find suitable candidates for immunoprophylactic purposes. Efforts have also been made to develop vaccines that could confer broad cross-protection against multiple serotypes of FAdVs, as conceptualized based on previous studies [72,73,74]. In 2014, a team developed an inactivated oil emulsion vaccine for FAdV-4 and evaluated it to determine whether any cross-protective immunity was achieved in vaccinated chickens challenged with multiple FAdV serotypes, including their progenies [57]. Five serotypes of Korean FAdV field isolates (4, 5, 8a, 8b, and 11) were used in the cross-protection trial, and sera were collected from challenged groups to evaluate the existence of serotype-specific antibodies, alongside postmortem examinations to check lesions. The results demonstrated that the mono-serotype FAdV-4 vaccine could provide broad cross-protection against all five serotypes of FAdVs used in this study, not only in vaccinated chickens but also in their progenies with confirmed maternally derived antibodies. The coordinated efforts by the research and industry sectors to control FAdV-4 outbreaks through vaccination programs were fortunately fruitful at some level, as cases involving this serotype started to decline rapidly in 2017 [45].

### 2.3. FAdV Outbreaks in 2013–2021

The proportion and number of cases of FAdVs reported between 2013 and 2021 are presented in Table 1 and Figure 1. A significant shift in the prevalence of dominant FAdV serotypes was observed in an epidemiological survey that characterized Korean FAdV strains isolated from clinical cases between 2013 and 2019 [45]. According to this report, it seemed evident that the introduction of inactivated FAdV-4 vaccines over the past several years was the cause behind this shift. The proportion of FAdV-4 related cases, the previous predominant serotype, decreased rapidly from 94% in 2016 to less than 20% in 2019. An interesting temporal epidemiological pattern followed by the declination of FAdV-4 cases was also noted. From 2017, FAdV-8b and -11 started to take over, with both serotypes gradually increasing until the sharp reduction in FAdV-11 in 2019, with FAdV-8b making up 80% of the proportion of field cases that year. Consequently, the number of HHS cases dropped dramatically, and most of the confirmed clinical cases after 2016 were IBH.

Although intervention by vaccination strategies for FAdV-4 has been successful, the emergence of new serotypes suggests that the current vaccines on the market do not exhibit cross-protection, specifically against FAdV-8b and -11. While there is evidence that cross-protectivity among different FAdV serotypes is somewhat valid under experimental conditions [57,75], it is rare in the field and unlikely to happen unless they are of the same species group [30,76]. The notable increase in FAdV-8b cases followed by the sharp drop in FAdV-11 cases in 2019 was also interesting. One theory behind this phenomenon is that commercial flocks may have naturally acquired immunity against FAdV-11 due to wild exposure. That is, the gradual increase in FAdV-11 cases in 2017–2018, followed by a rapid decrease in 2019, may reflect the temporal delay when naturally acquired immunity against FAdV-11 was building up within flocks until they could provide adequate protection that led to the permanent reduction in FAdV-11. It is also not hard to imagine that the acquired antibodies against FAdV-11, which are undisputedly imperative to preventing vertical transmission, were readily transferred from parental to progeny birds. Elevated herd immunity due to seroconversion as a response to natural exposure to viral pathogens is not unusual in poultry and has been noted in other reports [77,78]. However, this was certainly not the case for FAdV-8b, and it seemed apparent that this “naturally acquired immunity” for FAdV-11 did not help reduce FAdV-8b circulation among flocks. The poor cross-protectivity between FAdV-8b and -11 in the field was previously observed in Australia [30] and Canada [13,76]. Another notable finding from this report concerning the epidemiological landscape of FAdVs is the emergence of a distinctive phylogenetic cluster consisting of FAdV-8b strains that seem to have originated from strains circulating in China [79]. In, 2021, Park and colleagues also revealed genetic information of eleven Korean FAdV-8b strains isolated in 2019 from IBH cases [60]. As with previous reports, all strains were phylogenetically grouped with Chinese FAdV-8b strains, further strengthening the assumption that the newly emerging 8b strains in South Korea were derivatives of Chinese strains.

Unpublished reports of FAdV cases confirmed the further developments of FAdV-8b outbreaks in South Korea after 2019 (Supplementary Table S1 and Figure S1). In 2020, FAdV-8b made up to 64% (14/22) of confirmed FAdV cases, followed by FAdV-11 (27%, 6/22) and FAdV-4 (9%, 2/22). Although data for 2021 are still being collected, as of July 2021, 80% (8/10) of FAdV cases were confirmed as FAdV-8b. From the available data, it is clear that the continued emergence of FAdV-8b cases can be ascribed to the absence of vaccination intervention programs against this serotype over the past couple of years.

## 3. Current Status and Future Perspectives

**Figure 2:**
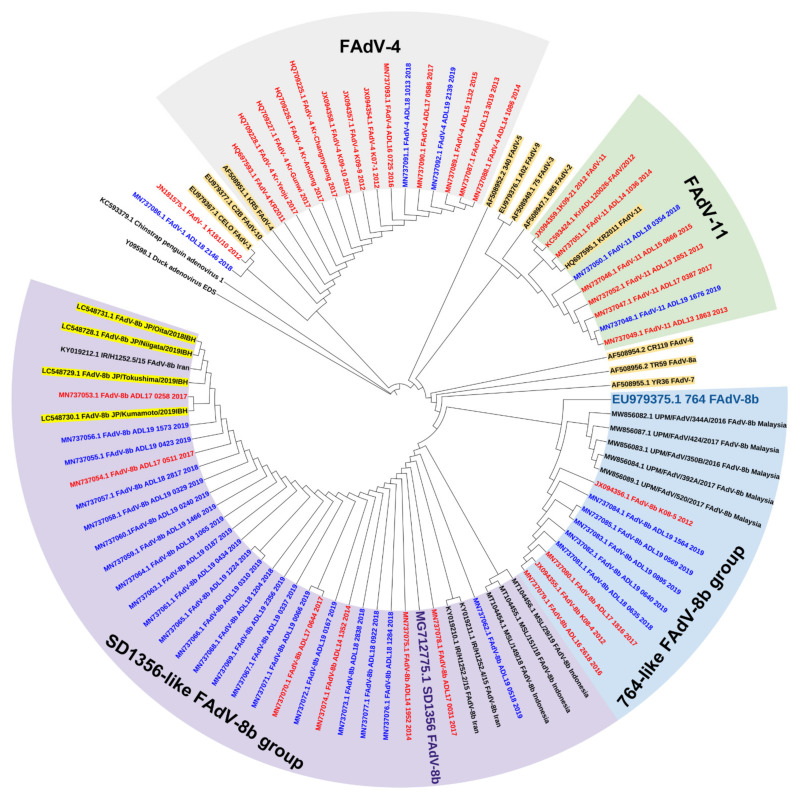
Phylogenetic analysis of Korean FAdV strains isolated from 2007 to 2019 based on the nucleotide sequences of the hexon loop1 gene. The genetic information of isolates was obtained from Genbank, and the tree was constructed by the maximum likelihood (ML) method with Mega version 10. Korean strains are indicated by colors based on the isolated year (red: before 2018, blue: 2018 and beyond). Reference strains are highlighted in brown. The Japanese FAdV strains isolated from 2018 to 2019 are highlighted in yellow.

Since Korean fowl adenoviruses were first molecularly characterized in the late 2000s, further investigations into their prevalence over the years have provided a comprehensive picture of the epidemiological landscape of this specific group of viruses (Figure 1 and Figure 2).

As virus spillovers are common across national borders, comparing the evolutionary status of FAdVs in neighboring regions would also provide a better understanding of the situation in South Korea. Epidemiological investigations in China revealed that FAdV-4 was the most dominant serotype circulating in the field, and its dominance persists to this day [80,81,82]. Similarly, in South Korea, the nationwide application of inactivated FAdV-4 vaccines in China also caused a shift in prevalence patterns, but with different characteristics [83]. Alongside FAdV-4 cases, the number of FAdV-11 cases also declined, indicating some cross-protective activity provided by FAdV-4 vaccines, which was disparate from the situation in South Korea when FAdV-11 cases soared after widespread application of FAdV-4 vaccines, showing little to no cross-protectivity. However, the rapid emergence of FAdV-8b after the reduction in FAdV-4 and 11 was a common trend between the two countries. Notably, the discovery of new types of FadV-8b strains in China has recently been reported [79,84], some of which share close genetic relationships with the new predominant FadV-8b strains in South Korea. For instance, the FAdV-8b SD1356 strain isolated from China in 2019 shares high genetic similarities with the recent Korean FAdV-8b isolates. This strain and the Korean isolates have been grouped together into a newly emerging phylogenetic cluster [45,60]. Furthermore, the FAdV-8b strains from the new cluster share similar pathogenic properties with the SD1356 strain, which are likely derivatives of this strains [79].

A similar situation was observed in Japan. Most of the recent Japanese FAdV-8b strains isolated between 2018 and 2019 also shared high genetic similarities with the Chinese SD1356 strain and were grouped into the same cluster [47]. Moreover, this study confirmed that a new cluster of SD1356-like FAdV-8b strains was emerging from the old group consisting of strains similar to the reference 764 strain, which was identically observed in Korean reports [45]. The IBH strains isolated from the Japanese study also shared close phylogenetic relationships with Korean SD1356-like strains and were clustered together (Figure 2). The close genetic relationship between the Korean and Japanese FAdV-8b isolates and the SD1356 strain suggests the spillover of Chinese FAdV strains to these countries. Previous IBH outbreaks in Japan were primarily attributed to FAdV-2 [31,85], but now it seems apparent that a new trend regarding the epidemiological status of FAdVs is currently developing.

Recently, a series of recombination events that may have increased the fitness of FAdV strains regarding host adaptation were first officially confirmed, particularly in strains from FAdV species D and E [86]. Moreover, it seems that FAdV strains circulating in the same geographical sphere can serve as genetic sources and exchange genes, which results in natural recombination, although crossing genus boundaries is likely uncommon. Nonetheless, evidence of intraspecies gene exchange involving FAdV-E strains demonstrated by this report further suggests a strong possibility that the recently circulating FAdV-8b strains that emerged into a new cluster in South Korea and Japan are likely products of recombination events between domestic strains and Chinese derivatives. However, as only the hexon loop1 gene was focused on for these strains in this review, analyzing complete genome sequences would provide a clearer picture of the alleged recombination events, leading to a much better comprehension of the FAdV status throughout the region.

The recently emerging FAdV-8b-associated IBH outbreaks in South Korea are not unique, and similar outbreaks have occurred worldwide; furthermore, the predisposing IBH serotypes do not seem to vary significantly according to geographic locations, at least in Asia. For instance, molecular characterization and phylogenetic studies of recent FAdV isolates from clinical cases in Malaysia revealed FAdV-8b as the predominant serotype behind IBH outbreaks among commercial broilers, similar to South Korea [48]. Interestingly, Malaysia does not have a history of FAdV outbreaks caused by serotypes other than FAdV-8b, which may be attributable to Malaysia being a self-sufficient producer that does not rely on international trade for poultry products, thus limiting the introduction of other serotypes [87,88,89]. That is, the Malaysian FAdV-8b cases may be ascribable to the “carry-over” or dissemination of viruses by wild birds; this may also apply to the Korean FAdV-8b cases that surged, although these assumptions require further corroboration. An increasing number of reports focus on wild birds functioning as potential reservoirs for FAdVs [37,38,39,90].

In neighboring Indonesia, FAdV-8b was one of the predominant serotypes [91], and FAdV-8b and -11 were confirmed as emerging causative agents for IBH in Iran [92]. Interestingly, all the Iranian and Indonesian FAdV-8b isolates from these studies were categorized into the SD1356-like FAdV-8b group, as the recent Korean and Japanese strains were, whereas Malaysian 8b isolates were classified as the 764-like FAdV-8b group (Figure 2). Since the Iranian and Indonesian FAdV-8b strains were isolated fairly recently, the possibility of them being recent derivatives or recombinants of the SD1356 strain cannot be ruled out. The rapid distribution of IBH-associated FAdV-8b strains in the past few years is becoming a worldwide phenomenon, including Asian and non-Asian countries [79,84,93,94].

Presently, there are no commercially available vaccines capable of countering the newly emerging FAdV-8b strains in South Korea, and it seems unlikely that the current FAdV vaccines could protect against future outbreaks concerning this group of viruses. This is especially unlikely when considering the incidence of vertically transmitted FAdV cases, as 195 out of 387 (50.1%) cases were roughly suspected of being ([45,55,58,59,60], Supplementary Table S2) based on the age of infection, which was below three weeks [22], comprising half of all documented cases. In addition, as most newly emerging FAdV-8b infections in 2020–2021 occurred in birds below that age, vaccines that could transfer adequate levels of antibodies from parental to progeny birds should be promptly developed and administered. Vaccines for the control and prevention of FAdVs have long been prioritized worldwide, with inactivated vaccines comprising the majority. Although inactivated vaccines are generally safe and practical, efforts towards developing alternative vaccine types are also becoming popular.

For example, live FAdV-8b vaccines have been used in Australia for to control IBH for some time, although results have not always been favorable, as the vaccines did not confer sufficient FAdV antibody titers in parental flocks, which led to the failure to protect progenies [30]. Attempts to attenuate virulent FAdV strains for exploring their potential use as live vaccines have been made [68,95], and the efficacy of some live FAdV vaccines has also been proven in recent prophylactic trials [96]. The natural advantages of using live FAdV vaccines would include the possible application via the oral route and more robust antibody responses elicited by live attenuated viruses.

Recombinant and subunit vaccines based on the structural proteins of FAdVs have also been successful in conferring suitable protection to vaccinated flocks. Since the first demonstration by Schachner and colleagues [35], most studies have focused on evaluating the application potential of FAdV-4 fiber proteins to prevent HHS, particularly in China, where FAdV-4-induced HHS outbreaks are still a major concern [34,97,98]. Inevitably, this concept has been extended to developing fiber-based subunit vaccines for IBH and has been reported to achieve vertical protection [36] and efficiently trigger B and T cell responses against FAdV-E IBH strains [99]. Lastly, hybrid or multivalent vaccines that provide broad-spectrum protection against IBH would also work as suitable vaccine candidates [100].

In conclusion, efforts to control FAdV-induced diseases can face numerous challenges due to their diverse set of serotypes with unique clinical characteristics and the emergence of variants worldwide. Nevertheless, conducting routine virus surveillance and monitoring programs in conjunction with vaccination intervention strategies is essential to minimize their occurrence and the associated economic implications.

## Figures and Tables

**Table 1:** Number of reported FAdV cases ^1^ in South Korea based on each serotype from 2007 to 2021.

Type	2007	2008	2009	2010	2011	2012	2013	2014	2015	2016	2017	2018	2019	2020	2021	Total
4	8	12	39	28	17	21	18	25	20	15	4	5	5	2	1	220
8b	-^2^	6	3	4	-	-	-	2	-	1	5	6	32	14	8	81
11	2	8	21	10	-	3	2	1	1	-	4	9	1	6	1	69
3	1	1	2	-	-	-	-	-	-	-	-	-	-	-	-	4
9	1	2	6	2	-	-	-	-	-	-	-	-	-	-	-	11
1	-	-	-	1	-	-	-	-	-	-	-	1	-	-	-	2
Total	12	29	71	45	17	24	20	28	21	16	13	21	38	22	10	387

^1^ Case numbers are based on published literature [45,55,58,59,60] and unpublished data (Supplementary Materials). ^2^ None reported.

## Supplementary Materials

The following are available online at https://www.mdpi.com/article/10.3390/v13112256/s1, Figure S1: Proportion of reported FAdV cases based on each serotype for the periods of 2007–2012 and 2020–2021. Table S1: Number of reported FAdV cases based on each serotype for the periods of 2007–2012 and 2020–2021. Table S2: History of reported FAdV cases based on each serotype for the periods of 2007–2012 and 2020–2021.
