# Supplementary material for: Historical Investigation of Fowl Adenovirus Outbreaks in South Korea from 2007 to 2021: A Comprehensive Review
Source: Viruses. 2021 Nov 10;13(11):2256. doi: 10.3390/v13112256 (PMC8621494; doi:10.3390/v13112256)
Supplement: Supplementary file 1 [file viruses-13-02256-s001.zip › Supplementary Table 1 (R3).pdf]

Supplementary table 1 Number of reported FAdV cases based on each serotype from 2007-2012 and 2020-2021.

Serotype [a]	2007	2008	2009	2010	2011	2012	2020	2021
**4**	5	7	18	17	17	21	2	1
**8b**	- [b]	1	-	3	-	-	14	8
**11**	1	3	4	3	-	3	6	1
**Total**	6	11	22	23	17	24	22	10

[a] Data here provided by courtesy of Avinext LTD, Cheongju, South Korea.

[b] Not reported
