# Supplementary figures and images for: Historical Investigation of Fowl Adenovirus Outbreaks in South Korea from 2007 to 2021: A Comprehensive Review
Source: Viruses. 2021 Nov 10;13(11):2256. doi: 10.3390/v13112256 (PMC8621494; doi:10.3390/v13112256)

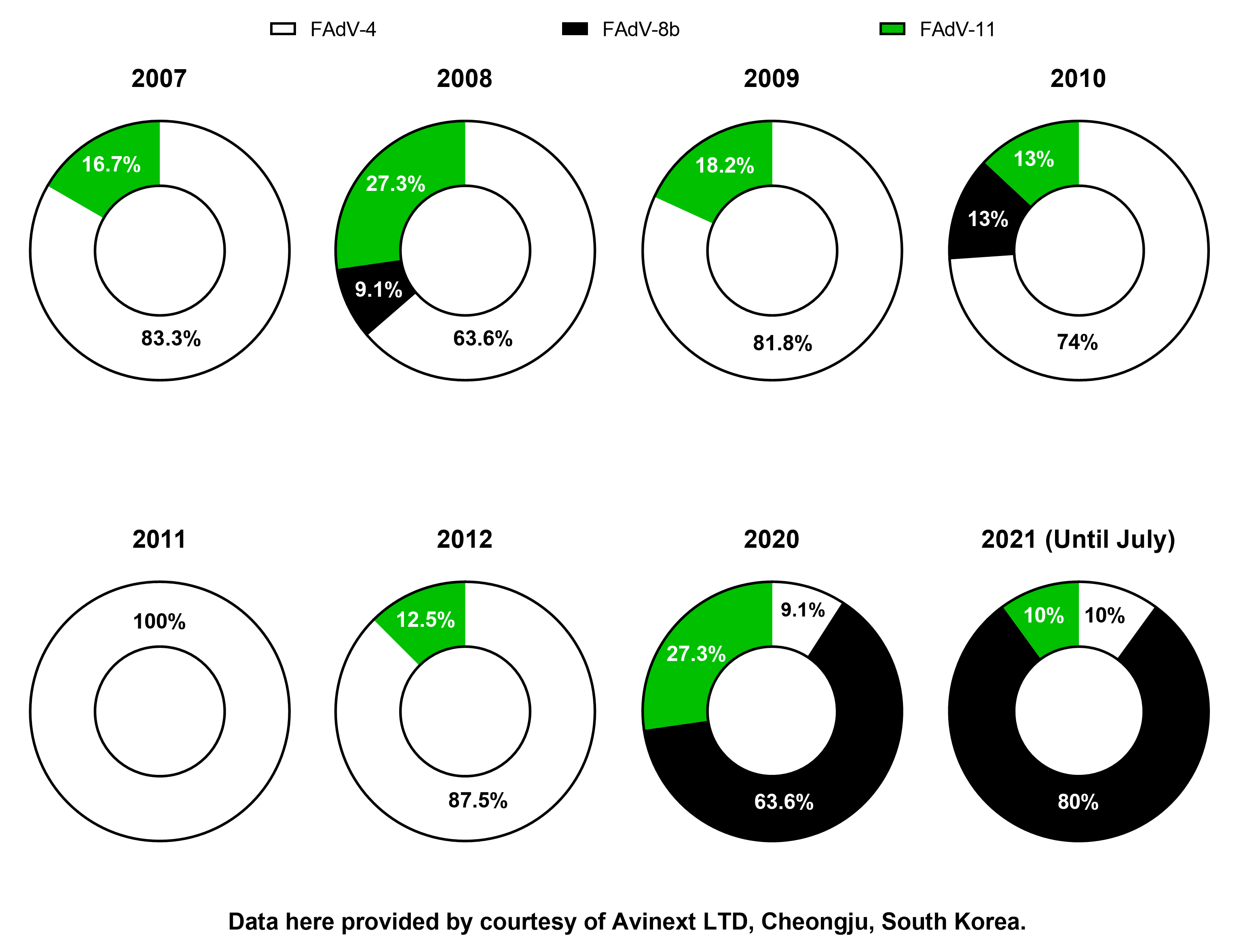

Supplement: Supplementary file 1 [file viruses-13-02256-s001.zip › Supplementary Figure 1 (R3).tif]
